# Supplementary material for: The interactome of intact mitochondria by cross-linking mass spectrometry provides evidence for coexisting respiratory supercomplexes
Source: Mol Cell Proteomics. 2017 Dec 8;17(2):216–32. doi: 10.1074/mcp.RA117.000470 (PMC5795388; doi:10.1074/mcp.RA117.000470)
Supplement: Supplemental Data [file supp_RA117.000470_133922_0_supp_23978_fzffwf.zip › spectra_annotation/mito_DR_spectra_annotation/269-1-3-1-8-1.pdf]

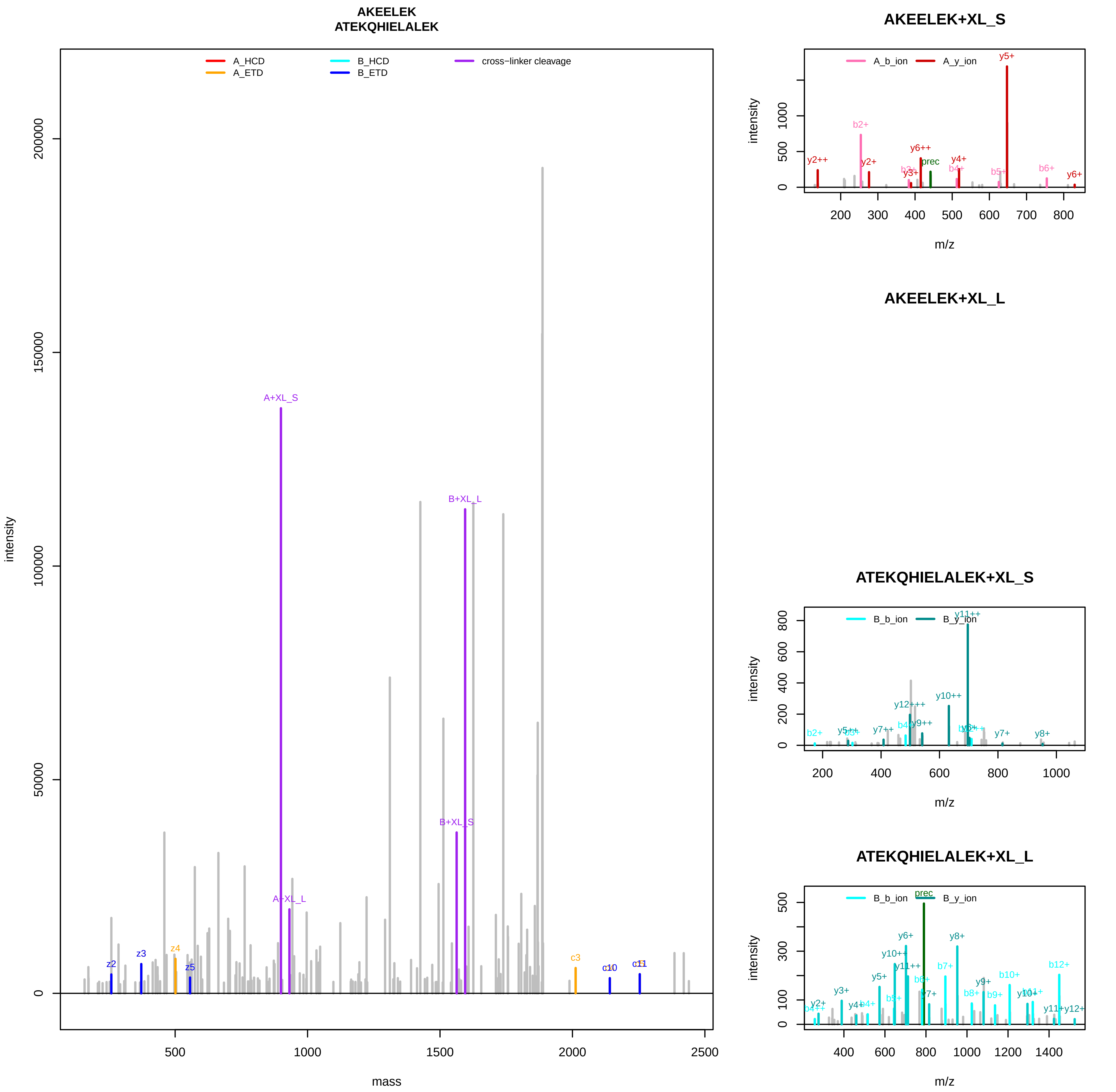
AKEELEK
ATEKQHIELALEK
A_HCD
A_ETD
B_HCD
B_ETD
cross−linker cleavage
intensity
mass
A+XL_S
A+XL_L
B+XL_L
B+XL_S
z2
z3
z4
z5
c3
c10
c11
AKEELEK+XL_S
A_b_ion
A_y_ion
y5+
b2+
y6++
y2++
y2+
prec
y4+
b4+
b5+
b6+
y6+
y3+
m/z
AKEELEK+XL_L
ATEKQHIELALEK+XL_S
B_b_ion
B_y_ion
y11++
y10++
y12+++
y9++
y7++
y5++
b2+
y7+
y8+
ATEKQHIELALEK+XL_L
prec
y6+
y8+
y10++
y11++
b7+
y5+
b12+
b10+
y9+
b8+
b9+
y3+
y2+
y4+
b4++
b5+
y7+
y10+
y11+
y12+
